# Role of Nrf2 in bone metabolism

**DOI:** 10.1186/s12929-015-0212-5

**Published:** 2015-10-29

**Authors:** Yong-Xin Sun, Ai-Hua Xu, Yang Yang, Jiliang Li

**Affiliations:** Department of Rehabilitation, The First Affiliated Hospital, China Medical University, No.155,North Nanjing Street, Heping District, Shenyang, 110001 China; Department of Biology, Indiana University Purdue University Indianapolis, Indianapolis, IN 46202 USA

**Keywords:** Nrf2, Osteoporosis, Osteoblast, Osteoclast, Reactive oxygen species

## Abstract

Nuclear factor erythroid 2-related factor 2 (Nrf2) is a transcription factor expressed in many cell types, including osteoblasts, osteocytes, and osteoclasts. Nrf2 has been considered a master regulator of cytoprotective genes against oxidative and chemical insults. The lack of Nrf2 can induce pathologies in multiple organs. Nrf2 deficiency promotes osteoclast differentiation and osteoclast activity, which leads to an increase in bone resorption. The role of Nrf2 in osteoblast differentiation and osteoblast activity is more complex. Nrf2 mediates anabolic effects within an ideal range. Nrf2 deletion suppresses load induced bone formation and delays fracture healing. Overall, Nrf2 plays an important role in the regulation of bone homeostasis in bone cells.

## Review

Mammalian bone tissue has a distinct composition and functions that set it apart from most other organs. There are a number of important functions carried out by bone. The skeleton’s primary function is to provide movement and support for the body that will withstand the daily challenges of weight bearing, to protect internal organs, and to maintain posture. Bones also act as a mineral reservoir, a growth factor and a fat repository. Ninety percent of calcium and 85 % of phosphate minerals are stored in bones [1]. Calcium and phosphate can be mobilized to sustain mineral homeostasis. Some of the most important regulators in skeletal metabolism include parathyroid hormone, calcitonin, Vitamin D, parathyroid hormone-related protein, insulin-like growth factor-1, and bone morphogenetic proteins [2–4].

Bone metabolism is a complex process that occurs throughout a lifetime. Bones are constantly changed through two processes: modeling and remodeling [5–7]. Bone modeling is the process of the formation of new bone that changes the shape of bones to adjust to forces in the environment. Bone remodeling is the process of the removal of old bone tissue that is being replaced with new tissue, which is essential for bone mineral homeostasis. Osteoblasts and osteocytes play two important roles in these two processes [8]. Osteoblasts secret bone matrix and control the mineralization of the bone. On the cell surface of osteoblasts are receptors for hormones such as vitamin D, estrogen, parathyroid hormone and parathyroid hormone-related protein. Osteocytes are thought to direct bone modeling and remodeling to accommodate mechanical strain and to repair damage [9]. They also activate osteoblasts to secrete RANK (Receptor Activator of Nuclear Factor κ B) - ligand that stimulates osteoclast differentiation. Osteoclasts and osteoblasts work together to remove old bone and add new bone [10]. Osteoclasts are activated by RANK-ligand to resorb old bone and release growth factors, such as TGF-beta, and to activate the migration of osteoblasts, which then deposit new bone matrix and orchestrate the mineralizing process.

Decreased levels of bone remodeling, or an imbalance between bone resorption and bone formation, have been linked to age-related bone loss, such as osteoporosis [11]. Osteoporosis is characterized by low bone mass and structural deterioration of bone tissue, leading to bone fragility and an increased susceptibility to fractures, especially of the hip, spine, and wrist. Osteoporosis occurs primarily as a result of normal aging, but can arise as a result of impaired development of peak bone mass or excessive bone loss during adulthood. Bone density scanners can measure bone mineral density and detect the disease. A report by the World Health Organization shows that fracture rates increase rapidly with age and the lifetime risk of fracture in 50 year-old women is about 40 % [12]. In 1990, there were 1.7 million hip fractures alone worldwide; with changes in population demographics, this figure is expected to rise to 6 million by 2050. The underlying molecular pathways leading to osteoporosis have not been well understood. In order to effectively select targets for therapeutic intervention of skeletal defects, it is essential to elucidate the basic molecular interaction in bone formation and resorption.

The following review will focus on how the transcription factor Nrf2 and its signaling cascade mediate differentiation, growth, and maintenance of osteoprogenitor cells and osteoclast precursors as well as their terminally differentiated lineage constituents. Also discussed are pathologies that arise when this pathway becomes disrupted.

### Overall roles of Nrf2

**Fig. 1:**
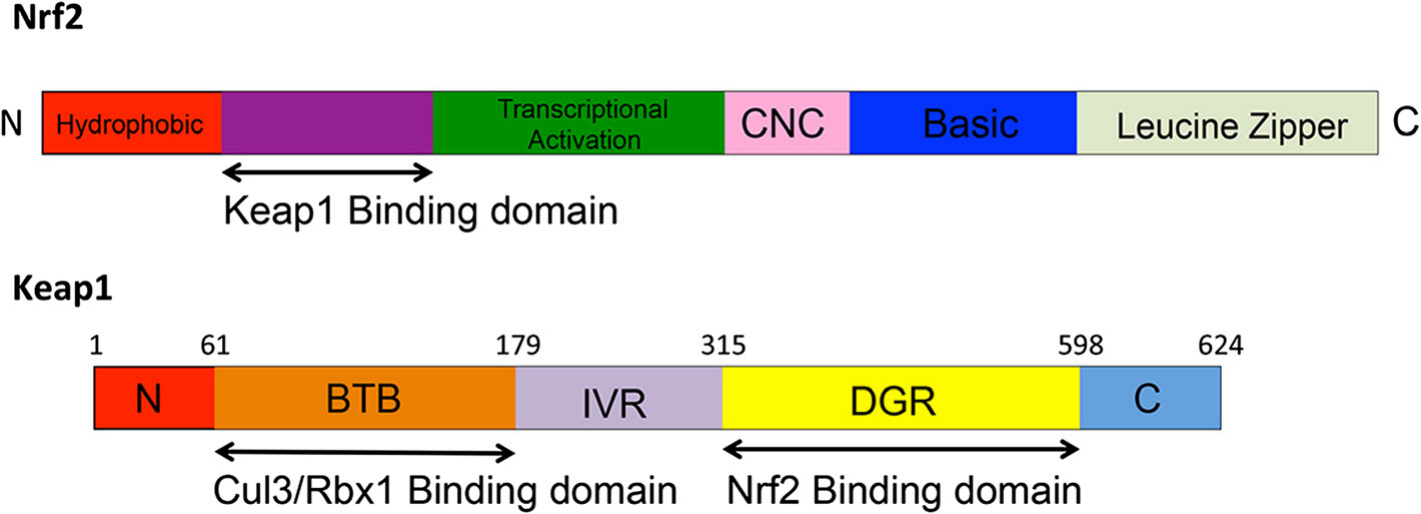
Structural domains of Nrf2 and Keap1. Nrf2 contains an N-terminal hydrophobic domain, followed by a Keap1-binding domain, transcriptional activation domain, CNC domain, and basic and leucine zipper domains. Nrf2, through its leucine zipper domain, heterodimerizes with small Maf or Jun proteins and binds to the ARE. Keap1, a homodimeric protein, retains Nrf2 in the cytoplasm. Keap1 functions as an adaptor for Cul3/Rbx1-mediated degradation of Nrf2. Keap1 with its BTB domain binds to Rbx1-bound Cul3, and its double-glycine repeat/Kelch (DGR) domain binds to Nrf2 and subsequently causes ubiquitination and degradation of Nrf2. BTB and DGR domains are separated by the intervening region (IVR)

Nuclear factor erythroid 2-related factor 2 (Nrf2) is a transcription factor expressed in many cell types, including osteoblasts, osteocytes, and osteoclasts. Nrf2 has been considered a master regulator of cytoprotective genes against oxidative and chemical insults. It belongs to a basic leucine zipper protein family [13], which comprises four members, namely Nrf1, Nrf2, Nrf3 and p45 NF-E2. Nrf1 and Nrf2 are ubiquitously expressed, whereas the expression of Nrf3 is restricted to placenta and liver, and NF-E2 is restricted to erythrocytes. Among these four members, Nrf2 is the main mediator of cellular adaptation to redox stress. The various domains of Nrf2 are shown in Fig. 1 [14]. It contains an N-terminal hydrophobic domain, Keap1-binding domain, transcriptional activation domain, CNC domain, and basic and leucine zipper domains. Nrf2 through its leucine zipper domain, heterodimerizes with small Maf or Jun proteins and binds to an antioxidant response element (ARE). Keap1 (Kelch-like ECH-associated protein 1), a homodimeric protein, retains Nrf2 for Cul3/Rbx1-mediated degradation of Nrf2.

**Fig. 2:**
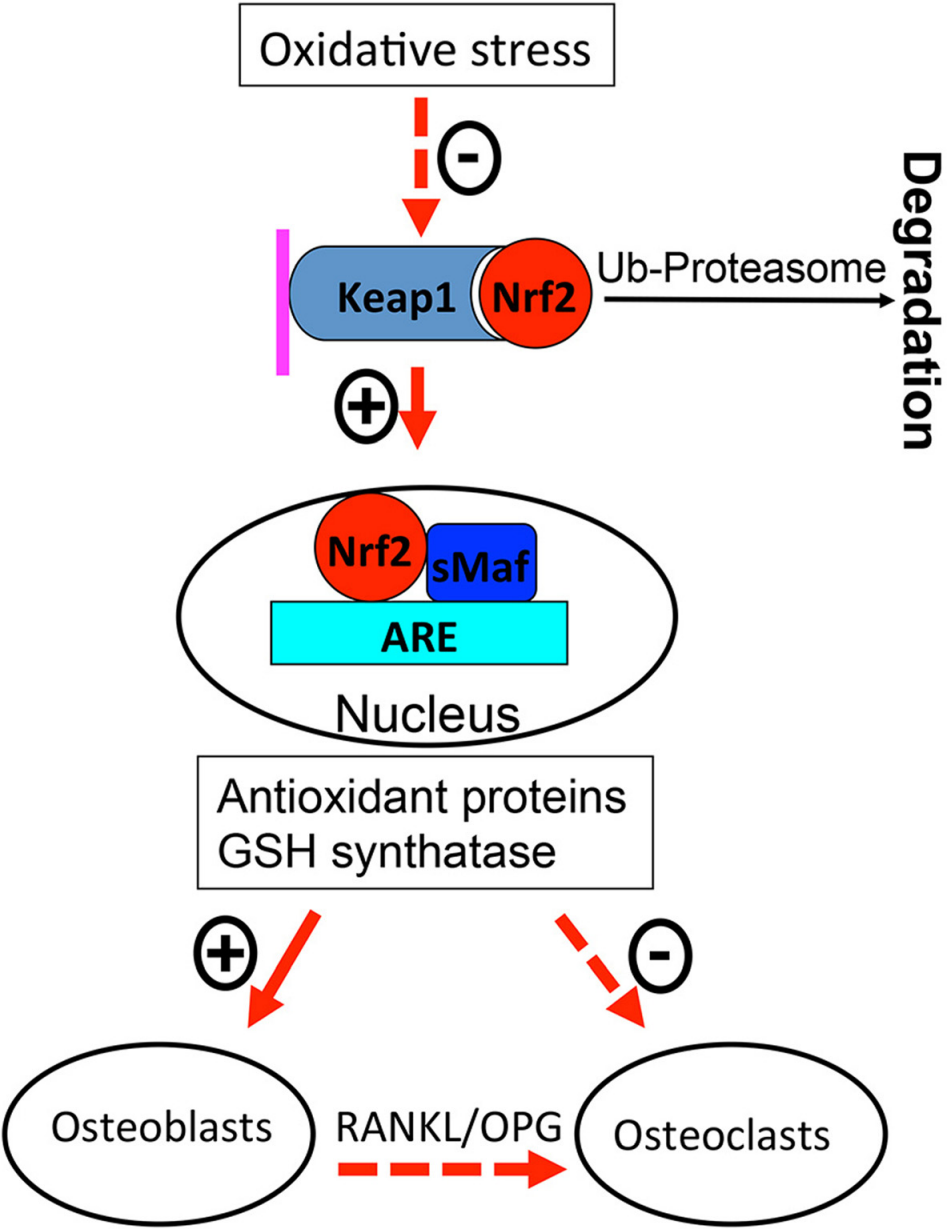
Activation of Keap1 and Nrf2 and their regulation in osteoblasts and osteoclasts. Oxidative Stress caused by aging and/or estrogen deficiency inhibits Keap1 and releases Nrf2 which subsequently migrates into the nucleus and activates antioxidant genes. Antioxidant proteins and GSH synthetase, which have positive effects on osteoblasts, negatively affect osteoclasts directly as well as indirectly via osteoblasts

Nrf2 is a key regulator of constitutive and inducible gene expression of phase II detoxifying enzymes and antioxidant proteins and is mainly expressed in metabolic and detoxification organs, such as the liver and kidney, as well as in organs that are continuously exposed to the environment, such as the skin, lungs, and digestive tract [15]. The molecular mechanisms of Nrf2 activation and transactivation activity have been revealed in recent years. As shown in Fig. 2, Keap1, a cytoplasmic actin-binding protein, tethers Nrf2 in the cytoplasm and represses the transactivation activity of Nrf2 under normal quiescent conditions [16]. Keap1 functions as a sensor protein of xenobiotics and oxidative stress. After exposure to either inducers of detoxifying enzymes or reactive metabolic intermediates produced by phase I bioactivation, Keap1 is modified, leading to the release of Nrf2 from Keap1, with the subsequent translocation of Nrf2 into the nucleus. Activated Nrf2 forms heterodimers with cofactors such as Small Maf proteins (sMafK and sMafG), and binds to an antioxidant response element (ARE), which exists in the regulatory regions of genes encoding detoxifying enzymes and antioxidant proteins, such as glutathione S-transferases (GSTs), NAD(P)H:quinone oxidoreductase (NQO) 1, and glutamate-cysteine ligase catalytic (GCLC) subunit [17–19], and transactivates the expression of these genes in many tissues, including bone and cartilage [20–22]. The ARE core sequence follows a pattern of TGACnnnGC [23]. Nrf2-ARE pathways are now recognized as one of the major cellular defense mechanisms against oxidative and xenobiotic stresses, and contribute to protection from various pathologies, including carcinogenesis, liver toxicity, respiratory distress, and inflammation [15]. Hence, Nrf2 has been considered as a novel molecular target for chemoprevention [21]. The importance of Nrf2 in many cytoprotective activities has been clearly illustrated through the use of Nrf2 knockout (KO, Nrf2^−/−^) mice [24]. The Nrf2 KO mice are more susceptible to pro-oxidant stimuli such as hyperoxia and many chemicals, suggesting that Nrf2 plays an important role in protecting a variety of tissues (lung, liver, kidney, central nervous system, etc.) from a wide array of toxic insults (electrophiles, reactive oxygen species, carcinogens, etc.).

### Roles of Nrf2 in bone homeostasis

The Nrf2 signaling pathway is emerging as an important factor in the regulation of bone metabolism. Oxidative stress induced by reactive oxygen species (ROS) increases with aging and/or estrogen deficiency in postmenopausal women and can adversely affect bone homeostasis and leads to skeletal fragility [25, 26]. In addition, antioxidant defenses are significantly decreased in postmenopausal osteoporotic women [27]. There is a possibility that the decrease in antioxidant systems in osteoporotic women is linked to the Nrf2 signaling pathway. Nrf2 deficiency leads to an increase in the intracellular ROS (Reactive Oxygen Species) level and a defect in the production of numerous antioxidant enzymes and glutathione in both osteoclast precursors and osteoblast progenitor cells [28, 29]. A normal balance between oxidants and antioxidants seems to be needed for the maintenance of a correct equilibrium between osteoblast and osteoclast activities, respectively [30]. Increased osteoclastic activity and decreased osteoblastic activity have been shown to be associated with an imbalance between oxidant and antioxidant status plasma biomarkers in postmenopausal osteoporosis [31]. Animal studies have shown that the deletion of Nrf2 in bone tissue leads to a lower bone mass as demonstrated by lower bone mineral density [32–35]. Without Nrf2 in mice, the bone strength of femurs and vertebral bodies was significantly lower in comparison to the wild type controls. Furthermore, load-driven bone formation was significantly inhibited in Nrf2-deficient mice compared to the controls. These data clearly show that Nrf2 plays an important role in bone homeostasis and mechanotransduction and further suggest that understanding of Nrf2 signaling in bone cells may provide new insights into the molecular mechanisms underlying bone homeostasis and discovery of new strategies to treat osteoporosis.

The lower bone mass and bone strength caused by Nrf2 deficiency result from an imbalance between bone formation and resorption. Histomorphometric measurement at the distal femurs shows that bone volume is significantly lower in Nrf2 KO mice than those of the controls [35]. Bone resorption surface was significantly greater and bone formation rate was significantly lower in Nrf2 KO mice than in the controls [35]. The decline in bone mass in Nrf2-deficient mice could be explained via bone remodeling, which includes sequential and coordinated activity of osteoclasts and osteoblasts to remove and restore old bone tissues continuously. In addition, a previous study using age- and sex-matched Nrf2^+/+^ and Nrf2^−/−^ mice show that a loss of functional Nrf2 increases radiation-induced bone loss [29]. These studies indicate that Nrf2 plays an important role in bone homeostasis.

### Roles of Nrf2 in osteoclasts

Nrf2 signaling is involved in regulation of osteoclast formation and activity. Osteoclasts are multinucleated cells derived from hematopoietic stem cells. The key inducer of osteoclast differentiation is RANK ligand primarily produced by osteoblasts. Exposure of RAW264.7 macrophages to RANK ligand lowers Nrf2/Keap1 ratio and leads to a decrease in the expression of Nrf2 dependent enzymes, which are in favor of ROS signaling. Overexpression of Nrf2 enhances RANK ligand induced elevation in the level of antioxidant enzymes and suppressed osteoclast differentiation. Deletion of Nrf2 suppresses antioxidant enzymes but elevates the intracellular ROS level in osteoclasts, subsequently increasing osteoclast number and stimulating osteoclast activity [28, 36]. This is consistent with a report showing that higher ROS induced more osteoclast differentiation [37]. Increased activity of p38 MAP kinase and NFATc1 induction, but not the NF-κB pathway, is responsible for the elevated level of ROS in Nrf2^−/−^ mice [28].

Consistent with previous studies [28, 29, 34, 36], our own data have shown that Nrf2 knockout results in an increase in osteoclast number in distal femurs and osteoclast surface in lumbar vertebrae, suggesting an increase in bone resorption in Nrf2^−/−^ mice [35]. Cell culture data show an increase in RANKL (RANK ligand) expression from Nrf2-deficient osteoblasts, which was also observed under stressful conditions, such as radiation [29]. These data suggest the increase in osteoclast number results from the higher expression of RANKL from Nrf2^−/−^ osteoblasts. On the contrary, overexpression of Nrf2 directly, or indirectly via inhibition of Keap1, decreases RANKL expression and reduces osteoclast number [36]. This represents an indirect effect of Nrf2 on osteoclast formation and activity. Further, Nrf2 may directly affect osteoclast activity via interfering with the actin ring. Mature osteoclasts have a sealing zone that consists of a ring of filamentous actin that is required for bone resorption [10]. Nrf2 deficiency promotes RANK ligand induced actin ring formation and bone resorption [28]. These data suggest that Nrf2 is necessary to maintain a normal range of bone resorption.

The role of Nrf2 in bone resorption has been focused on the osteoclast differentiation influenced by ROS and RANKL. Nrf2 activity in osteoclast progenitor cells and osteoclasts has not been well studied. Nrf2 regulates NFATc1 (Nuclear Factor of Activated T-cells, cytoplasmic 1) [34]. NFATc1 is considered as a master transcription factor in osteoclastogenesis [38]. We hypothesize that Nrf2 directly regulates osteoclastogenesis and osteoclast activity through interaction with NFATc1. In order to test this hypothesis, osteoclast- specific Nrf2 deficient mouse models should be studied.

### Roles of Nrf2 in osteoblasts

The role of Nrf2 in osteoblast differentiation and activity is quite controversial and may depend on factors, such as age, sex, genetic background, and physiological versus pathological conditions. Nrf2 is required for normal postnatal bone acquisition [33]. Our own data indicate that the lower bone mass of Nrf2^−/−^ mice in comparison with wild type controls not only results from an increased bone resorption but also from reduced bone formation, as shown by lower MAR (Mineral Appositional Rate) and decreased bone formation rate at distal femurs and lumbar vertebrae [35]. Deteriorated biomechanical properties of femurs and even lumbar vertebral bodies where bone mass was still not significantly decreased in Nrf2-deficient mice are also observed. These data are supported by another report that Nrf2-deficient osteoblasts lose their ability to differentiate and mineralize [29]. However, another study by Park et al. shows a higher bone density in Nrf2^−/−^ mice compared with the wild type controls [34]. In this study, 9-week-old Nrf2-deficient mice were analyzed, although the sex of the animals used was not described. There are possibilities that the sex and/or age as well as the genetic backgrounds would cause the discrepancy between the report by Park et al. and others.

The role of Nrf2 in osteoblast progenitor cells may be associated with the level of intracellular ROS. ROS is elevated in Nrf2-deficient stromal cells [29]. Oxidative stress caused by increased ROS has an inhibitory effect on osteoblast differentiation [39, 40]. On the other hand, low physiological amounts of reactive species may be necessary to promote osteogenesis. Bone morphogenetic protein 2 (BMP2) promotes differentiation of osteoblast progenitor cells to mature osteoblasts via ROS production by NOX4 (NADPH oxidase 4) [41]. Moreover, hydrogen peroxide is produced by differentiation osteoprogenitor cells and is required for mineralization and expression of osteogenic marker genes [42]. These data suggest that the relationship between osteoblastogenesis and Nrf2 or ROS is rather complex.

It seems that overexpression of Nrf2 causes deleterious effects in osteoblasts because overexpressing Nrf2 in MC3T3-E1 osteoblastic cells inhibits Runx2 [20, 34]. Runx2 is a master transcription factor regulating both embryonic bone development and postnatal osteoblastic function [43]. Endochondral and intramembranous bone formation is almost completely suppressed in mice deficient for Runx2, and chondrocyte maturation is arrested before the stage of hypertrophy [44, 45]. Based on this evidence, some believe that Nrf2 might inhibit osteoblastogenesis. As a matter of fact, many transcription factors in bone have narrow optimal expression ranges. For example, Runx2 must be maintained at optimal levels, as both Runx2 insufficient mice and Runx2 overexpressing animals are osteopenic [46, 47]. Perhaps the same is true of Nrf2, especially given its interactions with Runx2. Similar observations have been found in other tissues. For instance, mice with overexpression of Nrf2 by inactivation of the Keap1 gene died before weaning due to the hyperkeratotic lesions in the esophagus and stomach, which led to obstruction of the upper digestive tract [48]. Constitutively active Nrf2 impairs liver regeneration in mice due to delayed hepatocyte proliferation and enhanced apoptosis of hepatocytes after liver injury [49]. Constitutively active Nrf2 in keratinocytes causes skin abnormalities, including hair loss [50]. These studies suggest that Nrf2 generates beneficial effects in cells only within an ideal range.

The important role of Nrf2 in bone formation cells can also be demonstrated during fracture repair as well as in response to mechanical loading. Nrf2 expression is activated during fracture healing [51]. Bone healing and recovery of mechanical strength are retarded in the Nrf2 KO mice compared to the WT mice [51]. These changes may be caused by the reduction in vascular endothelial growth factor (VEGF) during fracture repair in Nrf2 KO mice. These data suggest that Nrf2 plays an essential role in bone regeneration.

### Roles of Nrf2 in mechanotransduction

Nrf2 signaling is involved in mechanotransduction. Nrf2 and NQO1 (NAD(P)H:quinone oxidoreductase 1) expression in calvarial osteoblasts increases in response to fluid shear stress [35]. In an axial ulnar loading study using Nrf2 KO mice [35], mineralizing surface and mineral appositional rate following the loading are significantly reduced in comparison to the controls, suggesting that osteoblast recruitment and activity are suppressed in response to mechanical loading. Deletion of Nrf2 significantly decreased NQO1 expression in calvarial osteoblasts. NQO1 is under transcriptional regulation by the Keap1/Nrf2 pathway, so upregulation of NQO1 mRNA or protein has been used extensively as a biomarker for Nrf2 activation [52, 53]. Further, upregulation of NQO1 protects the cells from oxidative damage due to the ability of NQO1 to reduce superoxide to hydrogen peroxide and generate antioxidant forms of vitamin E and co-enzyme Q [54, 55]. These data support observations that mechanical loading increases the oxidative stress in osteoblast-like cells [56] and that exercise can also lead to an increase in antioxidants in bone [57]. It is not clear if exercise induces oxidative stress and the antioxidant in parallel or if the antioxidant is a response to oxidative stress during exercise. It is reasonable to speculate that oxidative stress would be overproduced if the antioxidant system were impaired during mechanical loading. The enhanced oxidative stress may further disrupt the loading-induced bone formation via antagonizing Wnt molecules such as Wnt5a [35], a noncanonical Wnt ligand with anabolic effects on bone formation [58]. These data suggest an essential role of Nrf2 in mechanotransduction.

## Conclusion

Nrf2 plays an important role in the regulation of bone homeostasis in bone cells. The loss-of-function mutations of Nrf2 reduce bone mass and load-driven anabolic responses. So far the role of Nrf2 in bone homeostasis has been investigated using global Nrf2 deficient mouse models. However, it may be difficult to exclude the influence of other tissues, such as endocrine organs, on bone tissues and cells. In order to investigate the specific role of Nrf2 in different cell populations, studying osteoclast-, osteoblast- and osteocyte-specific Nrf2-deficient mouse models would give more insight into the important role of Nrf2 in bone homeostasis, mechanical properties, and load-driven bone formation.

## Abbreviations

Nrf2: Nuclear factor erythroid 2-related factor 2
RANK: Receptor activator of nuclear factor κ B
ARE: Antioxidant response element
Keap1: Kelch-like ECH-associated protein 1
GSTs: Glutathione S-transferases
NQO1: NAD(P)H:quinone oxidoreductase (NQO) 1
ROS: Reactive oxygen species
NFATc1: Nuclear factor of activated T-cells, cytoplasmic 1
MAR: Mineral appositional rate
NOX4: NADPH oxidase 4
